# Comparison of various pharmaceutical properties of clobetasol propionate cream formulations - considering stability of mixture with moisturizer-

**DOI:** 10.1186/s40780-020-0158-y

**Published:** 2020-01-30

**Authors:** Yoshihisa Yamamoto, Yoshinori Onuki, Toshiro Fukami, Tatsuo Koide

**Affiliations:** 1 Faculty of Pharmaceutical Sciences, Teikyo Heisei University, 4-21-2, Nakano, Nakano-ku, Tokyo, 164-8530 Japan; 2 Faculty of Pharmacy and Pharmaceutical Sciences, University of Toyama, 2630 Sugitani, Toyama, 930-0194 Japan; 3 Meiji Pharmaceutical University, 2-522-1, Noshio, Kiyose, Tokyo 204-8588 Japan; 4 Division of Drugs, National Institute of Health Sciences, 3-25-26, Tonomachi, Kawasaki-ku, Kawasaki, Kanagawa 210-9501 Japan

**Keywords:** Clobetasol propionate, Cream, Rheological property, Near infrared spectroscopy, Generic, TG-DTA, Mixture

## Abstract

**Background:**

The clobetasol propionate cream formulations (CLB_*Cr*_) belong to the “strongest” group, and are used widely. In addition, those formulations are often used as a mixture with moisturizer. Recently, we evaluated pharmaceutical properties of the CLB_*Cr*_ using near infrared (NIR) spectroscopy, and characteristic NIR spectra depending on the formulation were observed. In the present study, we attempted to evaluate the more diverse pharmaceutical properties of CLB_*Cr*_, including the stability of mixture of CLB_*Cr*_ and moisturizer.

**Method:**

Pharmaceutical properties of CLB_*Cr*_ were evaluated using from rheological characteristics, microscopic observation, dye permeability observations, electrical conductivity method, thermogravimetry-differential thermal analysis (TG-DTA) and near infrared (NIR) spectroscopy. Stability of mixtures of CLB_*Cr*_ and moisturizer were evaluated using from dye method and NIR spectroscopy.

**Results:**

The hardness of Dermovate® (DRM), Glydil® (GDL), and Myalone® (MYA) was greater than that of CLB_*Cr*_. High concentrations of white beeswax were considered the reason for the hardness of DRM and GDL. On the other hand, the hardness of MYA may be due to the presence of macrogol 6000. After storage of the cream formulations discharged from the tube at room temperature, mass reduction and attenuation of the peak reflecting water of NIR spectroscopy occurred in a time-dependent manner, except for GDL and MYA. Only GDL was shown to be a w/o-type formulation by dye and electric conductivity measurements, which suggested that this was the reason for the lack of changes in the mass or NIR spectrum of samples after storage. In the NIR spectrum of MYA, the peak reflecting water slightly increased in a time-dependent manner, suggesting the water absorption of macrogol 6000. TG-DTA provided curves indicating the presence of water in each formulation, except for MYA, which was consistent with water quantification previously reported. Finally, when mixing the CLB_*Cr*_ with a moisturizer, in any CLB_*Cr*_, the stability of the mixture with w/o-type moisturizer varies greatly depending on the each CLB_*Cr*_.

**Conclusion:**

Thus, even for cream formulations with the same active pharmaceutical ingredient, pharmaceutical properties and stability of mixture with moisturizer may different significantly.

## Background

Steroidal external formulations for skin application are used primarily for anti-inflammatory purposes and are classified into five groups according to their efficacy: strongest; very strong; strong; medium; and weak. The clobetasol propionate cream formulations (CLB_*Cr*_)  belong to the “strongest” group, and are used widely.

To provide information that cannot be obtained from interview forms issued by pharmaceutical companies to the medical field, we have evaluated the pharmaceutical properties of the external formulation on the skin. In particular, we evaluated the distribution of active and inactive pharmaceutical ingredients of alclometasone dipropionate ointments by using microscopic attenuated total reflection-infrared spectroscopy [1]. Furthermore, we also examined the quality of white petrolatum used in clobetasone butyrate ointments by using GC-MS [2]. The results indicated that two or more of the compared formulations had different pharmaceutical properties, even if they were classified as identical dosage forms. In contrast, a cream formulation was clearly distinguished from an ointment in the Japanese Pharmacopoeia 16 revision, and was defined as a “semi-solid formulation milked in oil-in-water (o/w) or water-in-oil (w/o) form to be applied to the skin”. It is clear that the excipient composition of cream formulations is more complicated than that of ointments, so a difference in pharmaceutical properties between the original and a generic formulation is more likely.

Near infrared (NIR) spectroscopy is frequently used for non-destructive analyses of food, agricultural products, and beverages [3–7]. NIR spectroscopy is also used in pharmaceutical sciences for applications, such as (i) qualitative validation of the components of dermatological formulations, ointments, or creams [8–11]; (ii) assessment of the degree of mixing of powders prepared by using a mortar and pestle, fine granules, and dry syrups [12]; and (iii) assessment of the distribution of the active pharmaceutical ingredient (API) and excipients in tablets by using microscopic NIR spectroscopy, which combines microscopy and NIR spectroscopy [13, 14]. Recently, we evaluated a CLB_*Cr*_ using NIR spectroscopy. Characteristic NIR spectra depending on the formulation were observed and it was revealed that the content of propylene glycol (PG) and water varied greatly depending on the formulation by GC-MS and the Karl-Fischer method [8]. These results indicated that the pharmaceutical properties varied greatly, depending on the formulation, even for formulations with an identical API.

The mixing of prescription compounds with steroidal formulations and moisturizers or base cording to patient needs is also common [15, 16]. The providing of instructions for mixing moisturizers with steroidal external formulations by physicians in Japan has improved compliance in pediatric patients [15]. Understanding the factors that influence the quality and stability of mixtures of external formulations is therefore very important for pharmacists. Nagelreiter et al. reported that the skin penetration of API is influenced by the type of cream base used [16]. Furthermore, numerous reports have described the influence of mixing on the release profile and skin permeation of API in external-application preparations [17–21]. Other studies have reported changes in the mixture formulation for a variety of combinations [22]. Such differences in pharmaceutical properties may affect the stability of the mixture with the moisturizer generally prescribed. In order to predict the stability of the mixture, it is necessary to understand not only the amount of water and excipients contained in the steroid cream formulation, but also the pharmaceutical properties from various viewpoints such as rheological properties, emulsion type, and microscopic properties. In the present study, we attempted to evaluate the more diverse pharmaceutical properties of CLB_*Cr*_, including rheological measurments, microscopic observation, dye permeability observations, electrical conductivity method, and thermogravimetry-differential thermal analysis (TG-DTA). Furthermore, we evaluate the stability of CLB_*Cr*_ and three type moisturizer (oil base, o/w-type and w/o-type) by NIR spectroscopy measurement in order to clarify the relationship between pharmaceutical properties of CLB_*Cr*_ and mixture stability.

## Methods

### Reagents

The original CLB_*Cr*_ analyzed was Dermovate® cream (lot. 14,016, GlaxoSmithKline K.K., DRM). The generic CLB_*Cr*_ analyzed were: Delspart® cream 0.05% (lot. 1412B, Ikeda medicine Industrial Co., Ltd.; DLS), Glydil® cream 0.05% (lot. SNXX, Sato Pharmaceutical Co., Ltd.; GDL), Mahady® cream (lot. A133S, Toko Pharmaceutical Industrial Co., Ltd.; MHD), Myalone® cream (lot. NZ02, Maeda Pharmaceutical Industry Co., Ltd.; MYA), and Solvega® cream (lot. 60,105, Hisamitsu Pharmaceutical Co., Inc.; SVG).

An oil based moisturizer, Propeto® (lot. 8Z031), a purified white petrolatum, was purchased from Maruishi Pharmaceutical Co., Ltd.

A heparinoid cream formulations Hirudoid® cream (lot. 5A05R, o/w-type; HRD_*OW*_) and Hirudoid® soft ointment (lot. 7EOLR, w/o-type; HRD_*WO*_) were purchased from Maruho Co., Ltd.

### Determination of rheological characteristics

A spread meter (Rigo Co., Ltd., Tokyo, Japan) was used to evaluate the spread of the cream. The diameter, D, of a 0.5 cm^3^ sample of ointment was measured after 5–200 s. The yield value S_0_ Pa was calculated from the formula of Ichikawa [23] by using D_∞_ cm at 200 s, the final measurement point [1].

In the formula, G is the acceleration due to gravity (980 cm/s^2^), P is the glass plate mass (460 g), and V is the volume of the sample (0.5 cm^3^).

1 $$ {S}_0=\frac{4.8 PVG}{\pi^2{D^5}_{\infty }} $$

Flow curves of shear rate versus shear stress were obtained by using a viscometer (TV-30; Toki Sangyo Co., Ltd., Tokyo, Japan). The temperature of the base plate was 30 ± 0.1 °C. The shear rate was varied from 0.38 to 9.58 s^− 1^.

### Microscopic observation

To characterize the microscopic features of the cream formulations, a small amount of the sample was applied to a microscope slide, covered with a cover slip, and observed by using an E-600-Pol polarizing microscope (Nikon Corporation, Tokyo, Japan) in reflection mode at 50×, 200×, 500×, and 1000× magnification.

### Dye method and electric conductivity method

An aqueous solution of 1.0% w/v methylene blue (lot. 331,829, Waldeck GmbH & Co. KG, MB), a water-soluble dye, and a liquid paraffin solution of 1.0% w/v Sudan III (lot. CTK0595, FUJIFILM Wako Pure Chemical Corporation), a fat-soluble dye, were prepared, and one drop was added to cream formulations spread out onto medicine packaging paper.

The resistance value was measured by using a digital multi meter CDM-6000 (CUSTOM corporation, Tokyo, Japan).

### CLB_*Cr*_ left at room temperature

Each of CLB_*Cr*_ (0.1 g) stored at room temperature (25 °C) was obtained after measurement of mass. After the set time has passed, the mass or NIR spectra of formulations were measured.

### Measurement of NIR spectra

The acquisition of NIR transmission spectra (optical path length: 0.2 mm) was determined by using a Spectrum One NTS spectrometer (PerkinElmer, Inc., Waltham, U.S.A.) equipped with an Omni Cell system used for mulls (Specac Inc., Cranston, U.S.A.) at a resolution of 8 cm^− 1^, employing 32 scans across the wavelength range 4000–8000 cm^− 1^. The NIR spectra of air was acquired as a background.

### TG-DTA measurements

The TG-DTA tests were performed using a simultaneous thermal analyzer (Thermo plus EVO2, TG-DTA8122, Rigaku, Japan). A 5 mg sample was placed into an aluminum crucible and then heated from 20 °C to 150 °C at a rate of 5 °C/min. The reference material was air.

### Preparation of mixtures consisting of CLB_*Cr*_ and moisturizers

Equal mass mixtures of CLB_*Cr*_ and moisturizer (Propeto®, HRD_*OW*_ or HRD_*WO*_) were prepared using a rotation/revolution type mixer, NRJ-250 (2000 rpm, 30 s; Thinky Co., Ltd., Tokyo, Japan). An aqueous solution of 1.0 w/v% MB and a liquid paraffin solution of 1.0 w/v% Sudan III were prepared, and 1 drop was added by dropper to the appropriate preparations prior to mixing. Centrifugation mixed samples were centrifuged at room temperature at 16500×g for 7 min. The condition of centrifugation was determined by previous report [24, 25].

### Statistical analysis

The significance of the differences between formulations was determined by using one-way analysis of variance (ANOVA) followed by a modified Fisher’s least-squares difference method. A *p* value of less than 0.05 was considered to be statistically significant.

## Results and discussion

### Test 1. Pharmaceutical properties of CLB_*Cr*_

#### Rheological properties

**Fig. 1:**
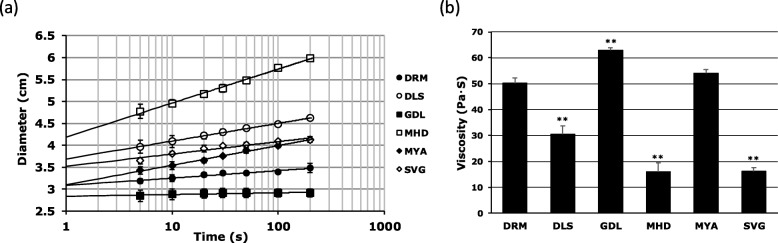
Rheological properties of CLB_*Cr*_. **a** changes in diameter of formulations in spread meter. Each point indicates the mean ± SD (*n* = 3). **b** viscosity values at 1.915 s^− 1^ of formulations in 30 °C. Each bar indicates the mean ± SD (*n* = 3).**, *p* < 0.01; significantly different from DRM

We conducted a spreadability test of the CLB_*Cr*_ marketed in Japan by using a spread meter. Figure 1a shows the plotted results, when the X axis was a logarithmic value of the spreading time and the Y axis is a sample diameter, in centimeters. The slope of the regression line, an indicator of spreading, of CLB_*Cr*_ obtained from these relationships ranged widely between 0.04 and 0.78 (Table 1). DRM was remarkably difficult to spread compared with other formulations except for GDL (0.17, Table 1). In contrast, the yield values of the formulations and the shear stress required to cause flow obtained from these relationships ranged widely, from 14 to 530 Pa (Table 1). The yield value of DRM was markedly higher than other formulations except for GDL (216.8 Pa, Table 1). These results suggest that the DRM formulation had the property of being difficult to flow after application on the skin and to spread around after the start of flow. In general, the cream formulations contain the basic ingredients of an oleaginous base, water, and a surfactant. The majority of the oleaginous bases used in creams was white petrolatum and fatty alcohol (e.g. cetanol and stearyl alcohol). As an alternative, white beeswax is used as oleaginous base for DRM (Table 2). In our previous studies, a single ointment containing 33% beeswax had low slope and high yield value (0.08 and 967.8 Pa, Additional file 1: Figure S1). Therefore, these results suggest that the hard property of DRM found in this study may be attributed to this excipient (Table 2). Beeswax has been reported to cause contact dermatitis [26], suggesting that changes to beeswax-containing cream formulation should be considered not only to adjust the feel, but also to avoid side effects.

**Table 1:** Slopes and yield values for CLB_*Cr*_ obtained from the spreadability test

Formulations	Slope	Yield value (Pa)
DRM	0.17 ± 0.09	216.8 ± 34.3
DLS	0.41 ± 0.10^**^	52.1 ± 1.1^*^
GDL	0.04 ± 0.03^*^	527.7 ± 81.6^**^
MHD	0.78 ± 0.07^**^	14.2 ± 2.7^**^
MYA	0.45 ± 0.10^**^	91.8 ± 7.0^**^
SVG	0.28 ± 0.03	93.2 ± 5.3^**^

Values are the mean ± SD (*n* = 3)

*, *p* < 0.05, **, *p* < 0.01; significantly different from DRM

**Table 2:** Pharmaceutical information of CLB_*Cr*_ used in this study

Formulations	Base	PG	^a^PG content (%)	^b^Water content (%)
DRM	white bees wax, cetostearyl alcohol	+	30.6 ± 2.5	26.2 ± 1.7
DLS	white bees wax, lanolin, liquid paraffin	+	2.1 ± 0.3	24.7 ± 1.8
GDL	white bees wax, white petrolatum, liquid paraffin, cetanol, microcrystalline wax	+	1.2 ± 0.1	32.8 ± 1.0
MHD	white petrolatum, cetanol	+	1.8 ± 0.3	46.7 ± 1.1
MYA	stearyl alcohol, macrogol 6000	+	26.1 ± 1.8	1.3 ± 0.5
SVG	white petrolatum, stearyl alcohol, liquid paraffin	–	–	55.8 ± 0.9

^a^; Propylene glycol content is presented as the mean ± SD (n = 3) by GC-MS cited in a recent manuscript^8)^

^b^; Water content is presented as the mean ± SD (n = 3) by the Karl-Fischer method cited in a recent manuscript^8)^

In all the generic CLB formulations except GDL (i.e. DLS, MHD, MYA, and SVG), the slope values of each formulation were significantly higher than DRM and the yield values were significantly lower than DRM (Fig. 1a; Table 1). In GDL, significantly lower slope and higher yield value than DRM were shown (Table 1).

Viscosity measurements by viscometer at 30 °C showed that, except for GDL and MYA, the viscosity values of the generic cream formulations were significantly lower than DRM (Fig. 1b). On the other hand, the value of GDL was significantly higher than DRM. The cause of the high viscosity of GDL is considered to arise from both the white beeswax and microcrystalline wax contained in this formulation (Table 2).

MYA had higher slope and lower yield value compared to those of DRM in the spreadability test, but the viscosity value of MYA was slightly higher than that of DRM (Fig. 1; Table 1). These results indicated that the viscosity after flow of MYA is equivalent to that of DRM and GDL, although MYA has greater fluidity than the formulations in the standing state. Macrogol 6000 and stearyl alcohol are contained in MYA (Table 2). Macrogol ointment, a mixture of macrogol 4000 and macrogol 400 in a mass ratio of 1:1, has the property of not spreading easily like a simple ointment compared with other classical bases (slope: 0.14, yield value: 508.2 Pa, Additional file 1: Figure S1). From these results, the rheological properties of MYA may be partially dependent on macrogol 6000. These results indicate that the rheological properties of CLB_*Cr*_ are very diverse. Such diversity is not observed at least in betamethasone butyrate propionate and betamethasone valerate cream formulations (Additional file 3: Table S1).

#### Microscopic observation

**Fig. 2:**
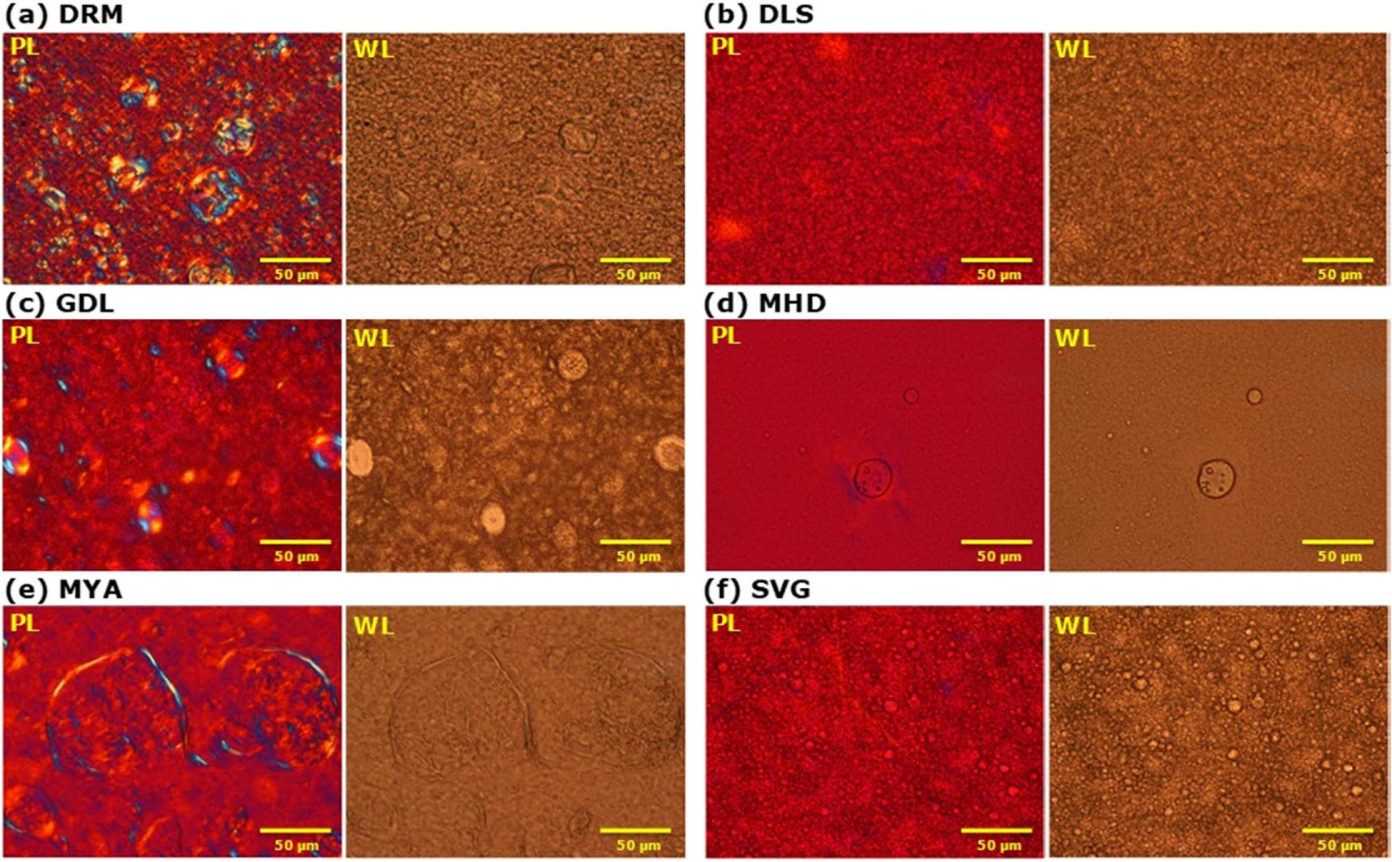
Microscopic images of CLB_*Cr*_ ((**a**) DRM, (**b**) DLS, (**c**) GDL, (**d**) MHD, (**e**) MYA and (**f**) SVG).; magnification: ×500. PL; polarized light, WL; white light

As the rheological characteristics of the CLB_*Cr*_ were found to differ, the microscopic properties of these formulations were observed by using a polarizing microscope. A dispersoid image of the continuous phase observed within the field of view varied widely depending on the formulation (Fig. 2; magnification × 500; see Additional file 2: Figure S2 for other magnifications). In DRM, GDL, and MYA, high-viscosity formulations, an image in which a dispersoid with a diameter of approximately 20 to 100 μm was dispersed in the continuous phase was observed. In addition, in MHD, dispersoids of approximately 20 μm in diameter were slightly recognized in the field of view. The rheological properties of such formulations are believed to be approximately dependent on the nature of the continuous phase (i.e. the water phase), suggesting that MHD exhibited the lowest viscosity (Fig. 1). Although the yield value of SVG was equivalent to that of MYA (Table 1), the viscosity was significantly lower (Fig. 1). Generally, in an emulsion, droplets interact with each other and then form a three-dimensional structure in the continuous phase. To make the emulsion flow, shear stress required to break the interactions is needed in addition to the shear stress to cause the continuous phase to flow. That is, the sum of those stresses corresponds to the yield value. After an emulsion starts to flow, its internal structure is gradually destroyed. The destroyed structure can be reconstructed by the removal of shear stress and there is a time lag before completion of the reconstruction, conferring thixotropic properties to the emulsions [27]. MYA is a special cream formulation that contains macrogol 6000, but not water. In contrast, SVG is a typical emulsion formulation in which fine dispersoids disperse. Therefore, it is considered SVG has clear thixotropic properties and low viscosity in flow conditions, as determined by the viscosity measurement.

#### Dye method and electric conductivity method

**Fig. 3:**
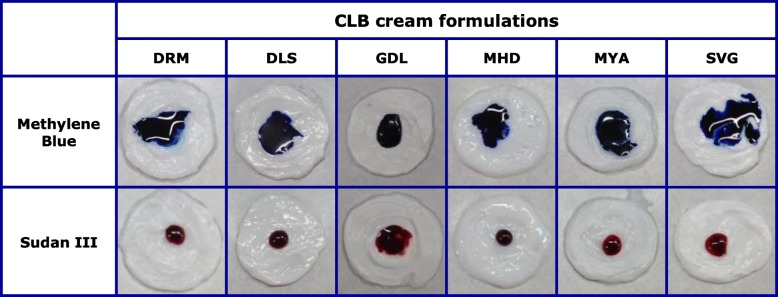
Dye permeability of CLB_*Cr*_

With the exception of GDL, penetration into the inside of methylene blue, but not Sudan III, was observed. In contrast, penetration into the inside of Sudan III, but not methylene blue, was observed only in GDL (Fig. 3). In addition, only the electrical resistance value of GDL exceeded the measurement limit of 60 MΩ (Table 3). These results indicated that five CLB_*Cr*_, including the original formulation, were o/w-type formulations, whereas GDL was w/o-type emulsion. Moreover, the electrical resistance value of MYA was higher than other o/w-type creams (22.4 MΩ, Table 3). The cause of this was considered to be the macrogol 6000 contained in MYA, which is a water-soluble base, instead of water (Table 2). The water content of this formulation was notably lower than the other o/w-type formulations [8]. Thus, it was revealed that the emulsion types may not necessarily match, even for cream formulations with the same API. By the way, betamethasone butyrate propionate and betamethasone valerate cream formulations are all o/w-type. Although there was some variation in the PG concentration in betamethasone butyrate propionate cream formulation, the water content in betamethasone butyrate propionate and betamethasone valerate cream formulations were similar (Additional file 4: Table S2).

**Table 3:** Electrical resistance values and emulsion type of CLB_*Cr*_

	DRM	DLS	GDL	MHD	MYA	SVG
Resistance value	502 ± 85 KΩ	482 ± 196 KΩ	> 60 MΩ	130 ± 22 KΩ	22.4 ± 2.3 MΩ	193 ± 85 KΩ
Emulsion type	o/w	o/w	w/o	o/w	o/w	o/w

Resistance values are represented as the mean ± SD (*n* = 3)

Emulsion type was determined comprehensively by the dye method (Fig. 3) and electrical conductivity method

#### NIR spectra and mass of CLB_Cr_ left at room temperature

**Fig. 4:**
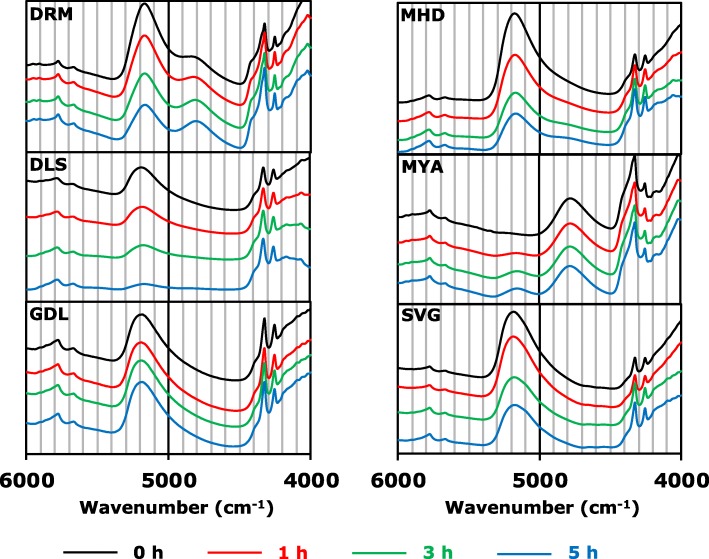
NIR-spectra of CLB_*Cr*_ stored at room temperature when removed from the tube

**Fig. 5:**
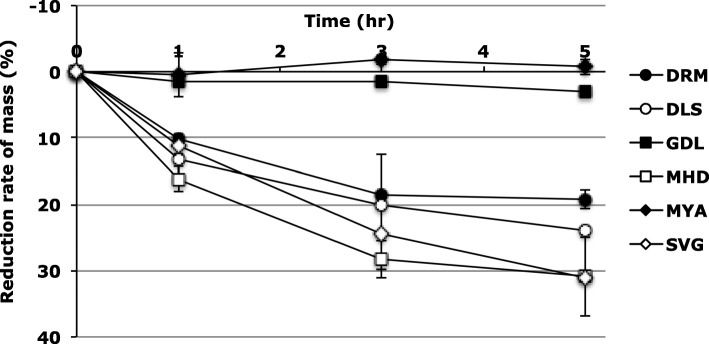
Rate of mass reduction of CLB_*Cr*_ stored at room temperature when removed from the tube (1–5 h). Each point indicates the mean ± SD (*n* = 3)

The peaks at approximately 4300 cm^− 1^ and 5700 cm^− 1^ derived from the combination and first overtone of hydrocarbons [28, 29] were observed in the NIR spectra for all of the cream formulations examined in this study. Moreover, peaks at approximately 5200 cm^− 1^ derived from the combination of a hydroxyl group [29, 30] were observed in all formulations except for MYA (Fig. 4), which indicated the presence of water. For only MYA, a peak at approximately 5200 cm^− 1^ was not observed, because this formulation contains little water (Table 2) [8]. A decrease in peak intensity at approximately 5200 cm^− 1^ in the NIR spectrum and mass for the o/w-type formulations, except for MYA, were obtained after storage at room temperature when removed from tube (Figs. 4, 5), suggesting the evaporation of water. For DRM, in addition to decreasing the peak intensity at approximately 5200 cm^− 1^, a clear peak at approximately 4800 cm^− 1^ appeared in a time-dependent manner. We reported that peak around the wavenumber reflects the presence of the alcoholic hydroxyl group [8]. As DRM contains 30% PG (Table 2), it is suggested that the decrease in water content leads to the occurrence of clear peaks derived from PG. In contrast, no change in the NIR spectrum and mass were obtained for GDL (Figs. 4, 5), suggesting the protection of water by the oily base, which is the continuous phase. For MYA, the increase in peak intensity at approximately 5200 cm^− 1^ in the NIR spectrum and mass was obtained after storage at room temperature when removed from the tube (Figs. 4, 5), suggesting the water-absorbing effect of macrogol 6000. Hence, it is considered that macrogol 6000 may be a factor in the rheological properties of MYA, as the large contribution of macrogol 6000 to the pharmaceutical properties of MYA was predicted from results in the present study. Hence, NIR was shown to be useful for the evaluation of the degradation of cream due to water evaporation.

#### TG-DTA measurement

**Fig. 6:**
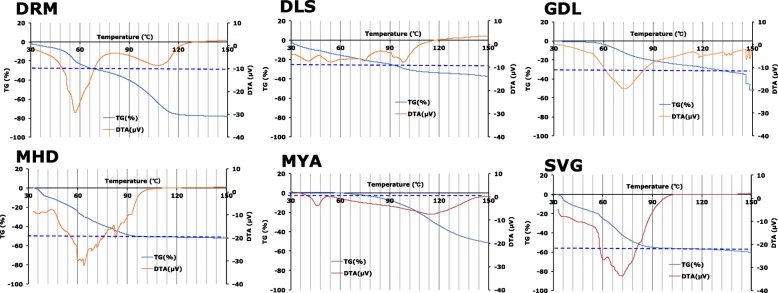
TG-DTA curve of CLB_*Cr*_. The dashed line indicates the water content determined by Karl-Fischer method (Table 2) in accordance with the TG scale

Mass reduction (TG) with an endothermic reaction (DTA) was observed for the temperature range of 50 °C–120 °C, except for DRM and MYA. As the mass reduction rate at approximately 100 °C and the water content of each formulation were nearly equal (Fig. 6, Table 2), this endothermic reaction was suggested to be mainly due to evaporation of water. Two endothermic peaks were observed up to 120 °C in the DTA-curve of DRM. Moreover, the decrease in mass of two phases was observed in this temperature range in the TG-curve. As the mass reduction rate of the first phase and the water content (Table 2) were consistent with each other, it suggests that the mass reduction associated with the endothermic reaction up to the first phase was due to the evaporation of water. There was an endothermic peak with no mass reduction at approximately 45 °C in MYA, which was suggested to be due to the melting of macrogol 6000. As no mass reduction was observed up to 80 °C, this TG-DTA result shows that this formulation contains little water (Table 2). In DRM and MYA, a gradual endothermic peak with mass reduction was observed at approximately 120 °C. This endothermic peak was considered to be due to the evaporation of PG, comprising 20–30% of DRM and MYA (Table 2). The mass reduction in GDL occurred at a higher temperature (approximately 60 °C) than other formulations, except for MYA, confirming that GDL as a w/o-type emulsion (Table 3); it was thought that the water evaporation was protected by the oil in the continuous phase. Thus, TG-DTA results also confirmed that the pharmaceutical properties of the CLB_*Cr*_ differed greatly, depending on the formulation.

### Test 2. Evaluation of the stability of mixtures of CLB_*Cr*_ and moisturizers

#### Mixture of CLB_Cr_ and oil base moisturizer

**Fig. 7:**
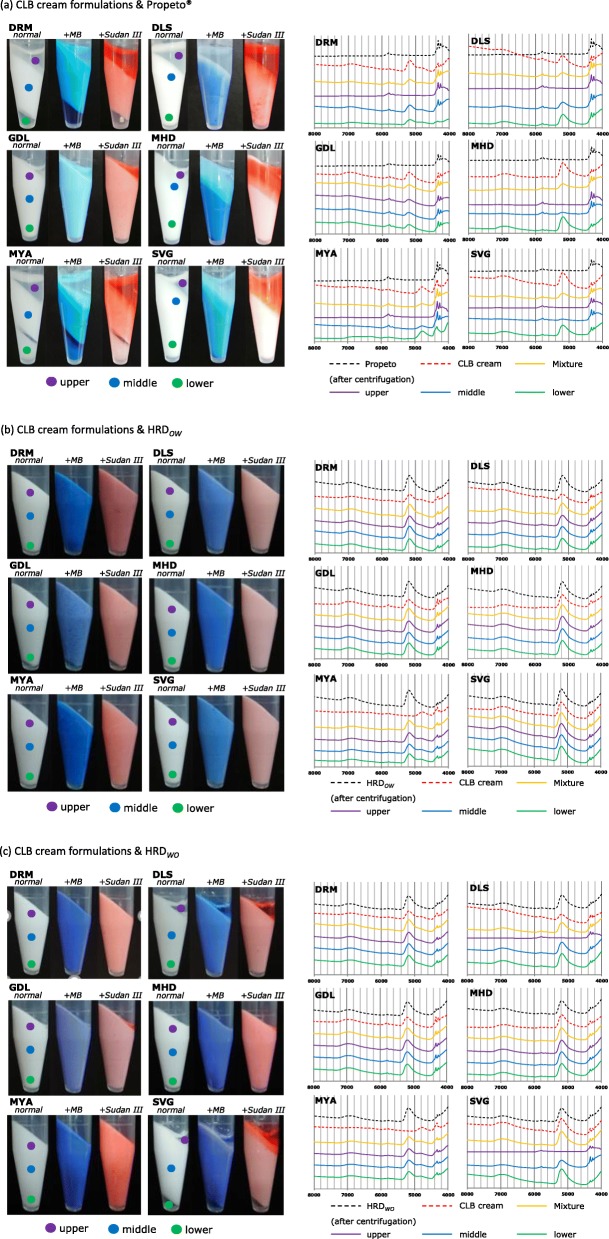
Appearance (*left*) and NIR spectra (*right*) of mixture consisting of CLB_*Cr*_ and moisturizer ((**a**) Propeto®, (**b**) HRD_*OW*_ and (**c**) HRD_*WO*_) after centrifugation . Appearance: One drop of methylene blue (MB) aqueous solution (1.0 w/v%) or Sudan III liquid paraffin solution (1.0 w/v%) was added prior to mixing. NIR spectra: The horizontal axis indicates the wave number (4000–8000 cm^− 1^). The vertical axis represents absorbance. Each spectra has been shifted vertically to improve visibility

When the mixture of CLB_*Cr*_ and Propeto® was centrifuged, separation into three layers was observed with preparations other than GDL. In the mixture previously added with MB or Sudan III, Sudan III and MB were localized on the upper layer side and the lower layer side, respectively, by centrifugation (Fig. 7a, *left*; Table 4). In the NIR spectra obtained by sampling the upper, middle and lower layers, the peak around 5200 cm^− 1^ reflecting the presence of water increased toward the bottom (Fig. 7a, *right*). These results indicate that the water is moving to the lower layer by centrifugation. Only the NIR spectrum of MYA the peak around 4800 cm^− 1^ increased toward bottom, but not peak around 5200 cm^− 1^ (Fig. 7a, *right*). MYA is a formulation that contains little water (Table 2)^8)^, suggesting that this change in the NIR spectrum reflects the movement of water-soluble alcohols such as PG and glycerin (Table 2)^8)^ to the lower layer. On the other hand, in GDL, although slight liquid was released in the upper part, almost no layer separation occurred. The continuous phase of GDL is an oleaginous component, suggesting that the mixture with Propeto® is more stable than other CLB_*Cr*_. These results indicate that mixing o/w-type CLB_*Cr*_ with oleaginous base is inappropriate.

**Table 4:** Changes in appearance when a mixture of CLB_*Cr*_ and moisturizer is centrifuged

Formulations	Propeto®	HRD_*OW*_	HRD_*WO*_
DRM	*θ*	ns	ns
DLS	*θ*	ns	*θ*
GDL	ns	ns	ns
MHD	*θ*	ns	ns
MYA	*θ*	ns	ns
SVG	*θ*	ns	*θ*

θ: layer separation is observed

ns: not separated

#### Mixture of CLB_Cr_ and o/w-type moisturizer

In the mixture of CLB_*Cr*_ and o/w-type heparinoid moisturizer, HRD_*OW*_, no layer separation was observed, and the distribution of MB and Sudan III were uniform (Fig. 7b, *left*; Table 4). The NIR spectra of the upper, middle, and lower parts were consistent (Fig. 7b, *right*). Since the cream formulations other than GDL were o/w-type (Table 3), it is suggested that the mixture was stabilized by matching the emulsion type. Interestingly, no layer separation was also observed in the mixture of GDL and HRD_*OW*_. GDL has the hardest properties among CLB_*Cr*_ (Fig. 1). We previously reported that there is a relationship between the maintenance of uniform water distribution and the viscosity of the base [25], suggesting that the high viscosity of GDL is also related to the stability of the mixture. In addition, it was considered that the balance of the oily base, water, and surfactant in the mixture was suitable for maintaining the emulsion.

#### Mixture of CLB_Cr_ and w/o-type moisturizer

Finally, in the mixture of CLB_*Cr*_ and w/o-type heparinoid moisturizer, HRD_*WO*_, layer separation was observed in DLS and SVG. And Sudan III and MB were localized on the upper layer side and the lower layer side (Fig. 7c, *left*; Table 4), respectively. In the NIR spectra obtained by sampling the upper, middle and lower layers, the peak around 5200 cm^− 1^ increased toward the bottom (Fig. 7c, *right*). The NIR spectra of the upper, middle, and lower parts matched for the four formulations that were not separated (Fig. 7c, *right*). The largest factor that caused layer separation in DLS and SVG would be the mismatch of the emulsion type. Although the DRM and MYA emulsion types were o/w-types, no layer separation was observed. It is considered that these hard properties contribute to the stability of the mixture (Fig. 1). Another o/w-type, MHD, showed no layer separation despite its low viscosity (Table 4). When the NIR spectra of MHD and HRD_*WO*_ were compared, the overall agreement was recognized (Fig. 7c, *right*). This fact reflects the good compatibility of both bases and may contribute to the stability of the mixture. Because GDL has a hard property as mentioned above and emulsion type match with HRD_*WO*_ only this formulation, suggesting that layer separation of the mixture did not occur.

Hence, these results suggest that the stability of the mixture with w/o-type moisturizer varies greatly depending on the each CLB_*Cr*_ and that the o/w-type moisturizer is the best choice generally when preparing a mixture with CLB_*Cr*_. In addition, the combination of o/w-type CLB_*Cr*_ and oleaginous base was found to be incompatible. A similar tendency has been obtained in studies using betamethasone valerate and betamethasone butyrate propionate cream formulations (Additional file 5: Table S3).

## Conclusion

The results of the present study indicated that cream formulations exhibit different pharmaceutical properties, although they contained the same API. At present, when a steroidal cream formulation was changed for therapeutic or economic reasons, pharmacists are required to select formulations to meet the constitution and desires of patients and with consideration of the pharmaceutical properties of the formulations.

## Supplementary information

**Additional file 1: Figure S1.** Changes in diameter of classical bases as determined by using a spread meter. Each point indicates the mean (*n* =3).

Microscopic images of CLB_*Cr*_. Magnification: ×50, ×200, and ×1000. PL: polarized light; WL: white light.

**Additional file 3: Table S1.** Slopes and yield values for betamethasone butyrate propionate and betamethasone valerate cream formulations obtained from the spreadability test.

**Additional file 4: Table S2.** PG content, water content and emulsion type of betamethasone butyrate propionate and betamethasone valerate cream formulations.

**Additional file 5: Table S3.** Changes in appearance when a mixture of betamethasone butyrate propionate and betamethasone valerate cream formulations and moisturizer is centrifuged.

## Data Availability

Not applicable.

## Abbreviations

API: Active pharmaceutical ingredient
CLB_*Cr*_: Clobetasol propionate cream formulations
DLS: Delspart® cream 0.05%
DRM: Dermovate® cream
GDL: Glydil® cream 0.05%
HRD_*OW*_: Hirudoid® cream
HRD_*WO*_: Hirudoid® soft ointment
MHD: Mahady® cream
MYA: Myalone® cream
NIR: Near infrared
PG: Propylene glycol
SVG: Solvega® cream
TG-DTA: Thermogravimetry-differential thermal analysis
