# Supplementary material for: Comparison of various pharmaceutical properties of clobetasol propionate cream formulations - considering stability of mixture with moisturizer-
Source: J Pharm Health Care Sci. 2020 Jan 30;6:1. doi: 10.1186/s40780-020-0158-y (PMC6990562; doi:10.1186/s40780-020-0158-y)
Supplement: Supplementary file 1 — Additional file 1: Figure S1. Changes in diameter of classical bases as determined by using a spread meter. Each point indicates the mean (n =3). [file 40780_2020_158_MOESM1_ESM.pptx]

## Slide 1
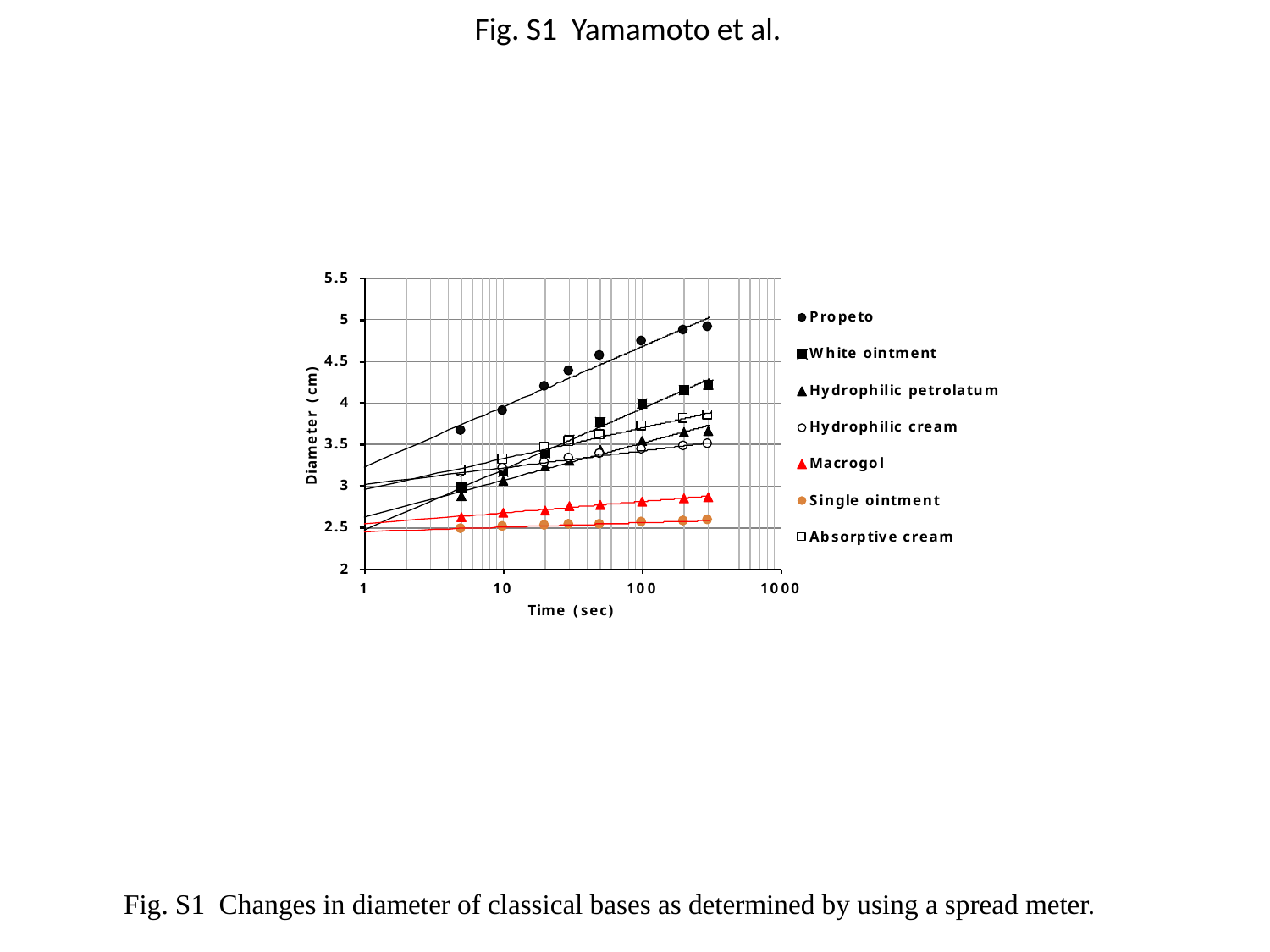

Fig. S1 Yamamoto et al.
Fig. S1 Changes in diameter of classical bases as determined by using a spread meter.
