# Supplementary material for: Comparison of various pharmaceutical properties of clobetasol propionate cream formulations - considering stability of mixture with moisturizer-
Source: J Pharm Health Care Sci. 2020 Jan 30;6:1. doi: 10.1186/s40780-020-0158-y (PMC6990562; doi:10.1186/s40780-020-0158-y)
Supplement: Supplementary file 2 — Microscopic images of CLBCr. Magnification: ×50, ×200, and ×1000. PL: polarized light; WL: white light. [file 40780_2020_158_MOESM2_ESM.pptx]

## Slide 1
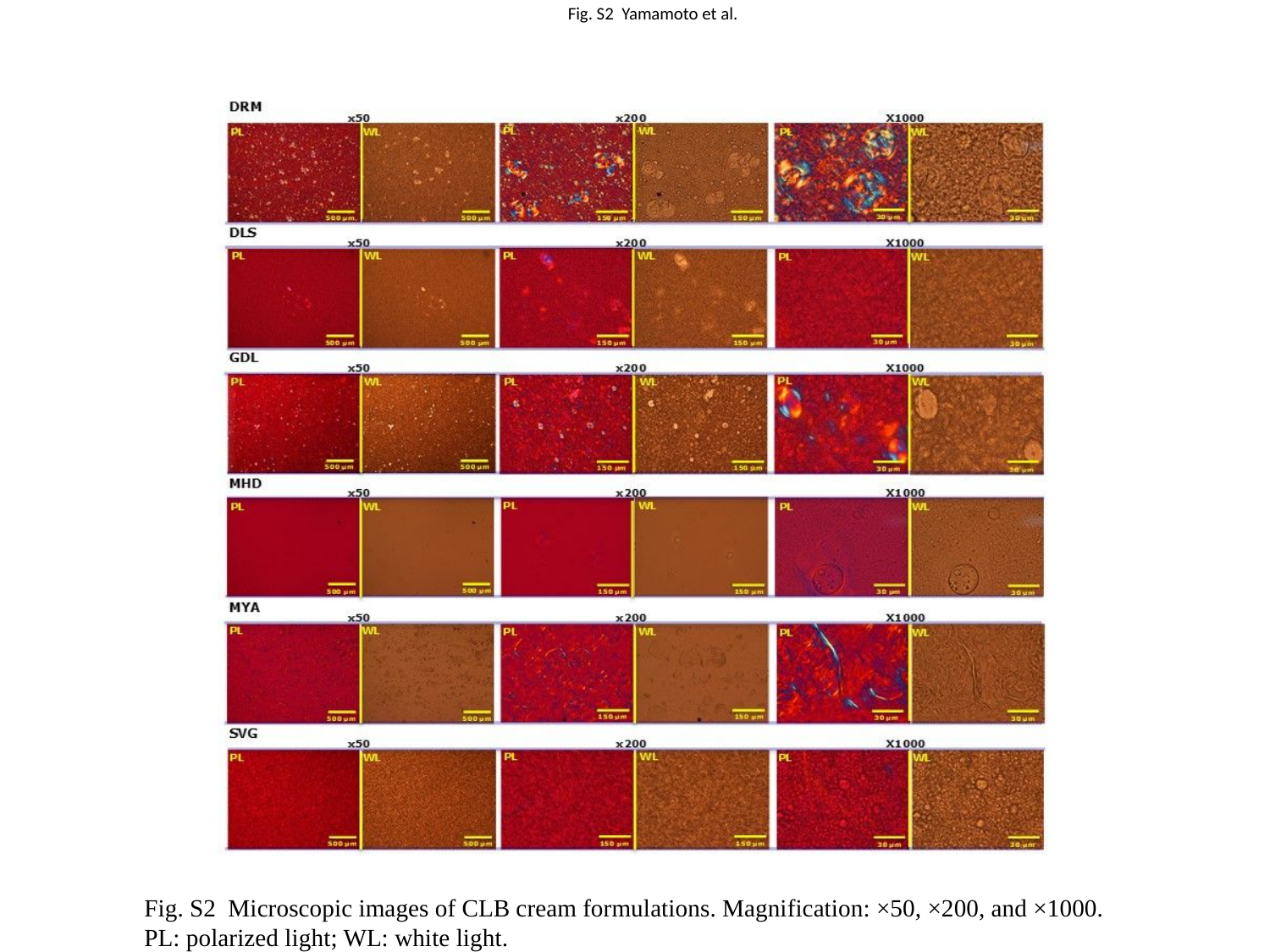

Fig. S2 Yamamoto et al.
Fig. S2 Microscopic images of CLB cream formulations. Magnification: ×50, ×200, and ×1000.
PL: polarized light; WL: white light.
