# Supplementary material for: Comparison of various pharmaceutical properties of clobetasol propionate cream formulations - considering stability of mixture with moisturizer-
Source: J Pharm Health Care Sci. 2020 Jan 30;6:1. doi: 10.1186/s40780-020-0158-y (PMC6990562; doi:10.1186/s40780-020-0158-y)
Supplement: Supplementary file 3 — Additional file 3: Table S1. Slopes and yield values for betamethasone butyrate propionate and betamethasone valerate cream formulations obtained from the spreadability test. [file 40780_2020_158_MOESM3_ESM.pptx]

## Slide 1
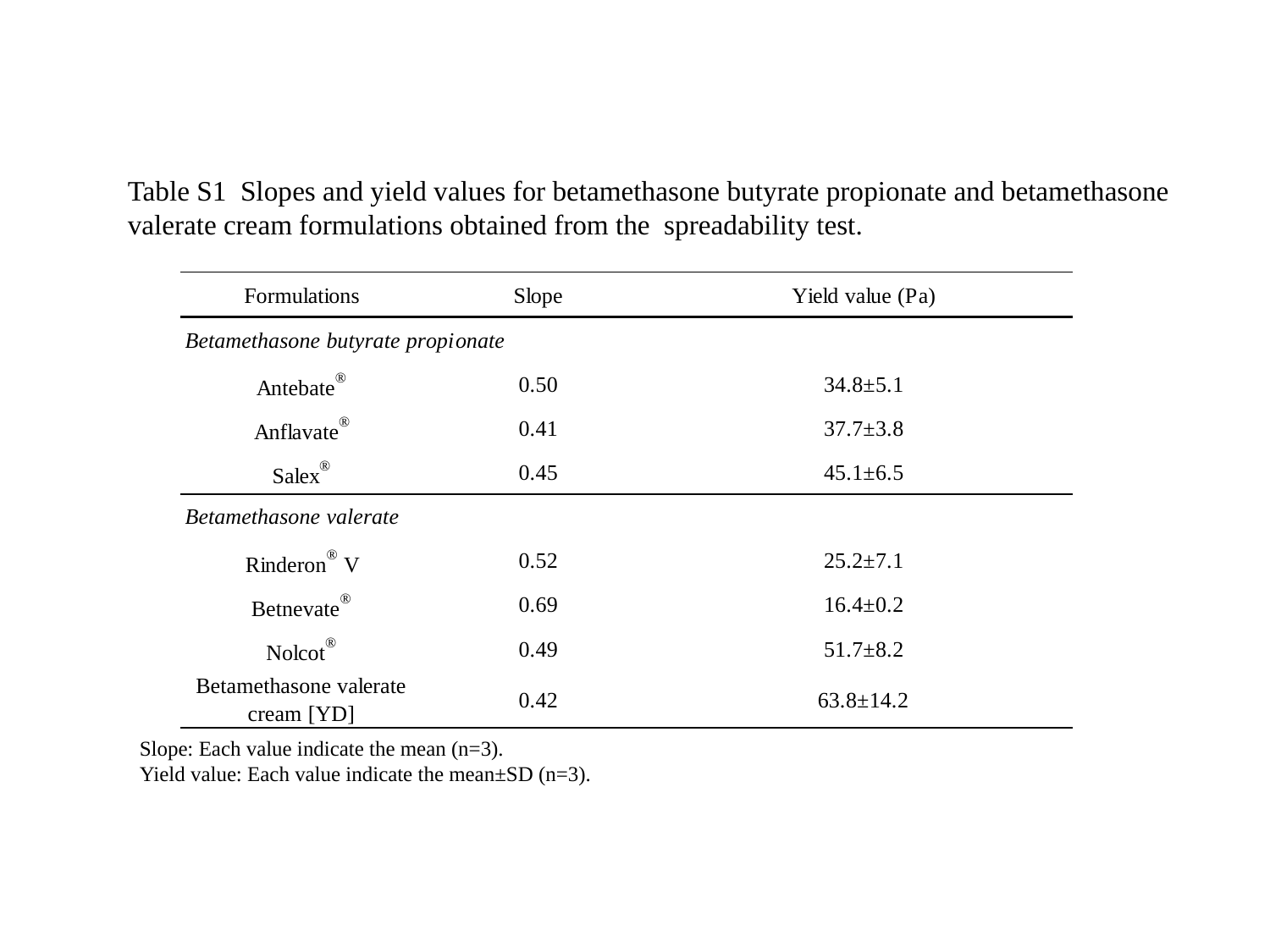

Table S1 Slopes and yield values for betamethasone butyrate propionate and betamethasone valerate cream formulations obtained from the spreadability test.
Slope: Each value indicate the mean (n=3).
Yield value: Each value indicate the mean±SD (n=3).
