# Supplementary material for: Comparison of various pharmaceutical properties of clobetasol propionate cream formulations - considering stability of mixture with moisturizer-
Source: J Pharm Health Care Sci. 2020 Jan 30;6:1. doi: 10.1186/s40780-020-0158-y (PMC6990562; doi:10.1186/s40780-020-0158-y)
Supplement: Supplementary file 4 — Additional file 4: Table S2. PG content, water content and emulsion type of betamethasone butyrate propionate and betamethasone valerate cream formulations. [file 40780_2020_158_MOESM4_ESM.pptx]

## Slide 1
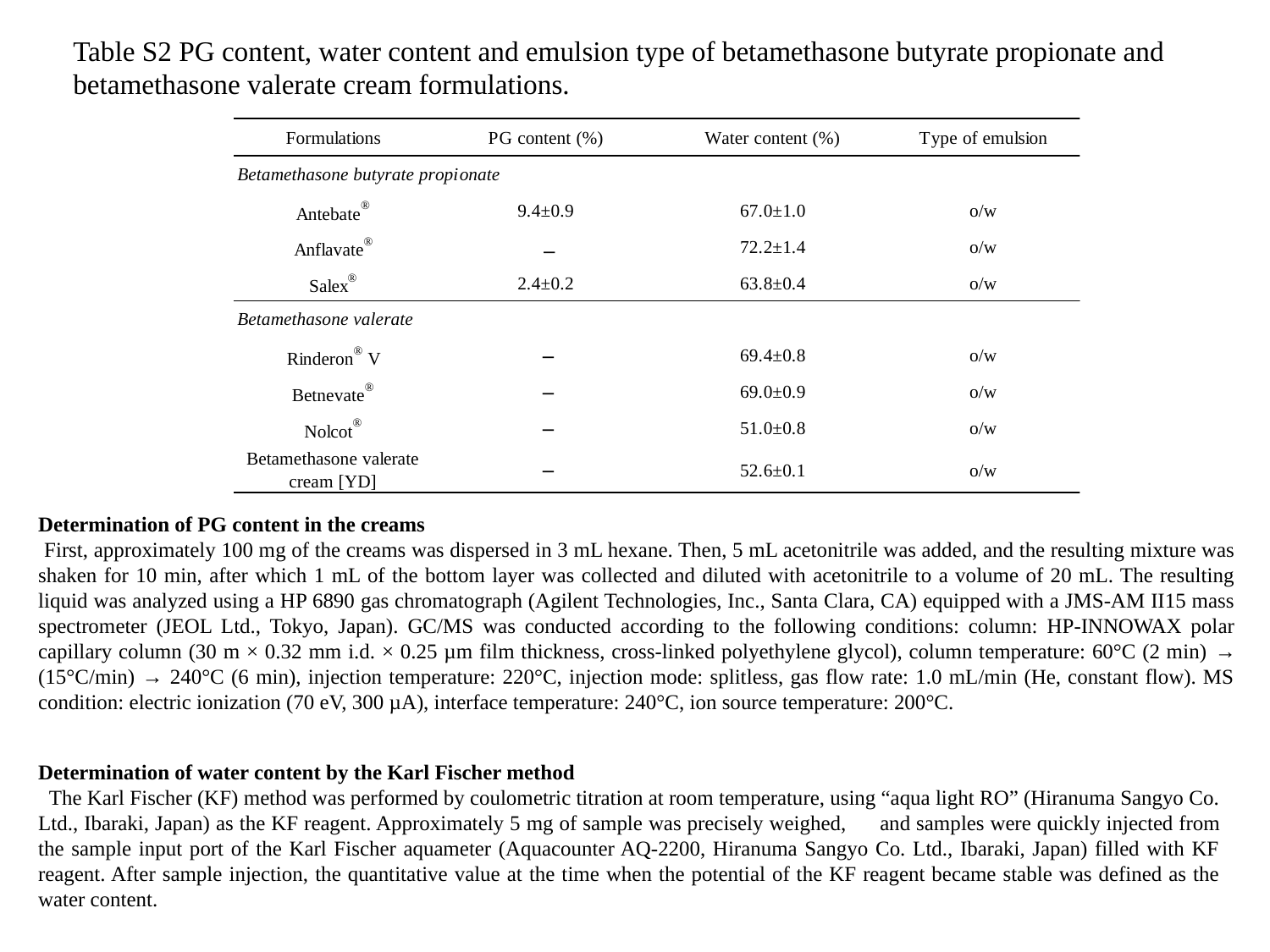

Table S2 PG content, water content and emulsion type of betamethasone butyrate propionate and betamethasone valerate cream formulations.
Determination of PG content in the creams
 First, approximately 100 mg of the creams was dispersed in 3 mL hexane. Then, 5 mL acetonitrile was added, and the resulting mixture was shaken for 10 min, after which 1 mL of the bottom layer was collected and diluted with acetonitrile to a volume of 20 mL. The resulting liquid was analyzed using a HP 6890 gas chromatograph (Agilent Technologies, Inc., Santa Clara, CA) equipped with a JMS-AM II15 mass spectrometer (JEOL Ltd., Tokyo, Japan). GC/MS was conducted according to the following conditions: column: HP-INNOWAX polar capillary column (30 m × 0.32 mm i.d. × 0.25 µm film thickness, cross-linked polyethylene glycol), column temperature: 60°C (2 min) → (15°C/min) → 240°C (6 min), injection temperature: 220°C, injection mode: splitless, gas flow rate: 1.0 mL/min (He, constant flow). MS condition: electric ionization (70 eV, 300 µA), interface temperature: 240°C, ion source temperature: 200°C.
Determination of water content by the Karl Fischer method
 The Karl Fischer (KF) method was performed by coulometric titration at room temperature, using “aqua light RO” (Hiranuma Sangyo Co. Ltd., Ibaraki, Japan) as the KF reagent. Approximately 5 mg of sample was precisely weighed,　and samples were quickly injected from the sample input port of the Karl Fischer aquameter (Aquacounter AQ-2200, Hiranuma Sangyo Co. Ltd., Ibaraki, Japan) filled with KF reagent. After sample injection, the quantitative value at the time when the potential of the KF reagent became stable was defined as the water content.
