# Supplementary material for: Comparison of various pharmaceutical properties of clobetasol propionate cream formulations - considering stability of mixture with moisturizer-
Source: J Pharm Health Care Sci. 2020 Jan 30;6:1. doi: 10.1186/s40780-020-0158-y (PMC6990562; doi:10.1186/s40780-020-0158-y)
Supplement: Supplementary file 5 — Additional file 5: Table S3. Changes in appearance when a mixture of betamethasone butyrate propionate and betamethasone valerate cream formulations and moisturizer is centrifuged. [file 40780_2020_158_MOESM5_ESM.pptx]

## Slide 1
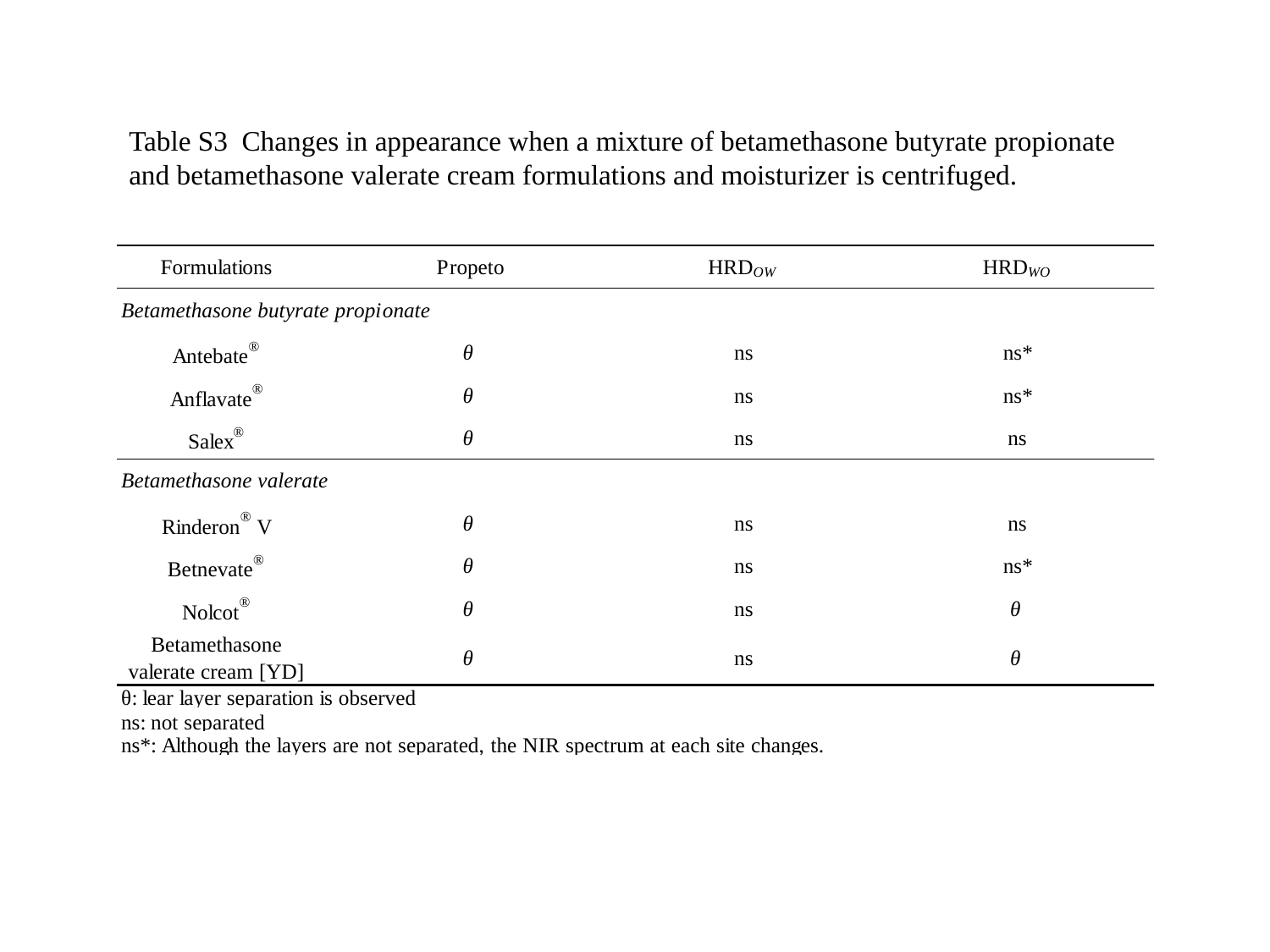

Table S3 Changes in appearance when a mixture of betamethasone butyrate propionate and betamethasone valerate cream formulations and moisturizer is centrifuged.
